# Artificial Intelligence: A New Tool for Structure-Based G Protein-Coupled Receptor Drug Discovery

**DOI:** 10.3390/biom15030423

**Published:** 2025-03-17

**Authors:** Jason Chung, Hyunggu Hahn, Emmanuel Flores-Espinoza, Alex R. B. Thomsen

**Affiliations:** 1 Department of Molecular Pathobiology, New York University College of Dentistry, New York, NY 10010, USA; jachung1107@gmail.com (J.C.); hh2763@nyu.edu (H.H.); ef2588@nyu.edu (E.F.-E.); 2 NYU Pain Research Center, New York University College of Dentistry, New York, NY 10010, USA

**Keywords:** structure-based drug discovery, AlphaFold, RoseTTAFold, computational docking, virtual screening, GPCRs

## Abstract

Understanding protein structures can facilitate the development of therapeutic drugs. Traditionally, protein structures have been determined through experimental approaches such as X-ray crystallography, NMR spectroscopy, and cryo-electron microscopy. While these methods are effective and are considered the gold standard, they are very resource-intensive and time-consuming, ultimately limiting their scalability. However, with recent developments in computational biology and artificial intelligence (AI), the field of protein prediction has been revolutionized. Innovations like AlphaFold and RoseTTAFold enable protein structure predictions to be made directly from amino acid sequences with remarkable speed and accuracy. Despite the enormous enthusiasm associated with these newly developed AI-approaches, their true potential in structure-based drug discovery remains uncertain. In fact, although these algorithms generally predict overall protein structures well, essential details for computational ligand docking, such as the exact location of amino acid side chains within the binding pocket, are not predicted with the necessary accuracy. Additionally, docking methodologies are considered more as a hypothesis generator rather than a precise predictor of ligand–target interactions, and thus, usually identify many false-positive hits among only a few correctly predicted interactions. In this paper, we are reviewing the latest development in this cutting-edge field with emphasis on the GPCR target class to assess the potential role of AI approaches in structure-based drug discovery.

## 1. Introduction

Proteins are essential for all cellular processes, mediating a diverse range of functions from catalysis, transportation, cell signaling, and structural support. The numerous functions of proteins stem from their specific amino acid sequences, which fold to form unique 3D structures. The intricate folding patterns are driven by a combination of both intra- and intermolecular forces, such as hydrogen bonding, hydrophobic effects, van der Waals forces, ionic interactions, and disulfide bonds [1]. These interactions work together to stabilize the protein’s structure, ensuring it folds correctly into its native conformation.

In addition to being important for understanding their function in biological processes, protein 3D structures provide critical insights into the potential for designing compounds that can bind to and modulate their function, thus offering a way to regulate entire biological pathways and processes [2,3]. Such compounds can act as starting points for the development of drugs to treat diseases [3].

Traditionally, information about how proteins are organized has been obtained by solving their structures experimentally. These efforts have provided us with an incredibly detailed understanding of proteins’ structure and function. However, solving the structures of proteins is not trivial and requires a large amount of resources and a significant time commitment. Thus, structure-based drug discovery has not been an effective strategy for finding drug-like molecules against targets where no structural information is available. Over the past decade, however, there has been a revolution in structural and computational biology. One of the most exciting developments in this respect has been the inclusion of artificial intelligence (AI)- and machine learning (ML)-based approaches to obtaining structural models of proteins [4,5]. In fact, these impressive approaches allow for highly accurate predictions of protein structures from as little information as primary protein sequences. Furthermore, prediction of ligand–protein interactions has also evolved significantly and as a result, we are approaching a point where early drug discovery of targets can be performed via computer in a highly resource- and time-efficient manner. In this article, we will review these late developments with a special focus on one of the most successful classes of drug targets: the superfamily of G protein-coupled receptors (GPCRs).

## 2. GPCR Structure and Function

GPCRs are cell surface receptors that regulate virtually all physiological functions, including cardiovascular, immunological, neurological, endocrine, and sensory functions, among others [6]. Common to all GPCRs is their structural arrangement, which includes an extracellularly located amino terminal followed by a seven α-helical transmembrane domain connected via intra- and extracellular loops, and an intracellularly located carboxy-terminal tail [7]. Within the receptor region accessible to the extracellular environment is the orthosteric binding pocket, which has specifically evolved to bind small organic molecules, peptides, lipids, carbohydrates, ions, and even larger proteins [7,8]. Due to these features, GPCRs are highly druggable, and currently about 33% of all prescribed drugs target them [6,7,9].

**Figure 1:**
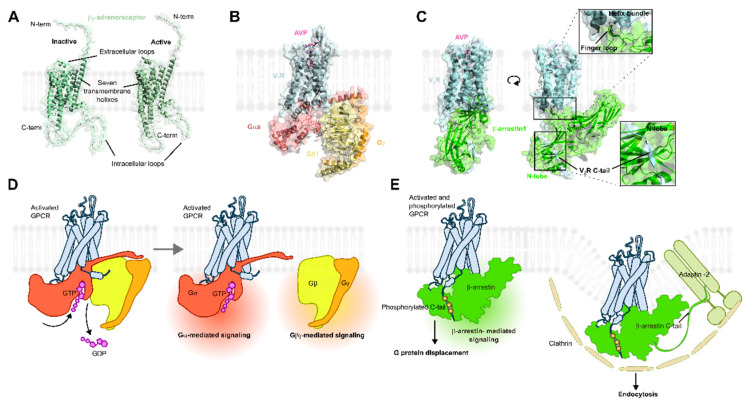
Structures and mechanisms of GPCR activation. (**A**) Structural models of inactive and active β_2_-adrenergic receptor (obtained from GPCRdb). (**B**) Structure of the argine-8 vasopressin (AVP)-bound vasopressin V2 receptor (V2R) in complex with the heterotrimeric Gs protein (PDB: 7bb6). (**C**) Structure of the AVP-bound V2R in complex with β-arrestin 1 (PDB: 7df9). (**D**) Mechanism of how ligand-bound GPCRs engage with heterotrimeric G protein, which promotes GDP to GTP exchange in the catalytic domain of the Gαs subunit. This event activates the G protein and causes the Gα to dissociate from Gβγ subunits. These activated G protein subunits initiate signaling cascades and ultimately change cellular behavior. (**E**) β-arrestin recruitment to an activated and phosphorylated GPCR uncouples G proteins from the receptor intracellular face, and thus, terminates G protein signaling. In addition, β-arrestins promote endocytosis of GPCRs by scaffolding adaptin-2 and clathrin and can induce its own signaling pathways.

Agonist engagement with the orthosteric binding pocket of GPCRs stabilizes an active receptor conformation, which has a high affinity for heterotrimeric G protein (Gαβγ) (Figure 1) [10]. The GPCR–G protein interaction leads to a GDP-GTP exchange within the Gα subunit, which causes activation and dissociation from the Gβγ subunits [11]. These activated Gα and Gβγ subunits then interact with and stimulate enzymes to produce second messengers (e.g., cAMP, IP3, DAG) as well as other signaling proteins. The net result of this event is the initiation of signaling cascades that ultimately lead to a cellular/physiological response. In parallel with these activities, the active conformation of the GPCR is recognized by GPCR kinases (GRKs), which phosphorylate the receptor at specific serine/threonine sites at the third intracellular loop and/or carboxy-terminal tail [12]. The phosphorylated receptor is then detected by arrestins, which translocate from the cytosol to the receptor at the cell surface. The formation of the GPCR–arrestin complex regulates G protein signaling as arrestins compete for the G protein-binding site at the receptor [13]. In addition, arrestins act as a scaffold for proteins involved in endocytosis, including clathrin and adaptin-2 [14,15]. Thus, receptor recruitment of arrestins also facilitates internalization of the receptor from the membrane into intracellular vesicular/tubular organelles called endosomes. Finally, arrestins themselves can initiate signaling events by associating with signaling proteins such as MEK/Raf/ERK and JNK3, and, thus, can modulate cellular behavior [16].

Synthetic ligands can be designed that either bind to the orthosteric binding pocket or other allosteric binding sites within GPCR to regulate its function [7,9]. Depending on the desired physiological effect, ligands can be designed to either stimulate (agonists), enhance (positive allosteric modulators), prevent (competitive antagonists), or decrease receptor activity (inverse agonists or negative allosteric modulators) [17,18]. In general, agonists, competitive antagonists, and inverse agonists convey their function via binding to the orthosteric binding site, whereas allosteric modulators bind to an allosteric binding site [6,9]. As GPCRs regulate most physiological functions [6], developing such ligands can be highly beneficial from a therapeutic point of view, and there are tremendous interests in discovering new leads that bind to specific binding sites. One of the most elegant strategies for achieving this is structure-based drug discovery.

## 3. Experimental Structure Determination

Current experimental methods to solve protein structures such as X-ray crystallography, NMR spectroscopy, and cryo-electron microscopy (cryo-EM) have created the baseline for uncovering the structures of over 100,000 proteins [19]. Of these proteins, around 1200 of them are GPCRs either by themselves with or without ligands or in complex with transducers such as heterotrimeric G proteins or arrestins “https://gpcrdb.org/structure/statistics (accessed on 1 October 2024)”. These structures are stored and logged in the Protein Data Bank (PDB) and prove the effectiveness and achievements of these techniques; however, these experimental methods are laborious and require a high level of expertise as well as major resources and a significant time commitment—and they do not guarantee success. This is particularly true for GPCRs.

**Figure 2:**
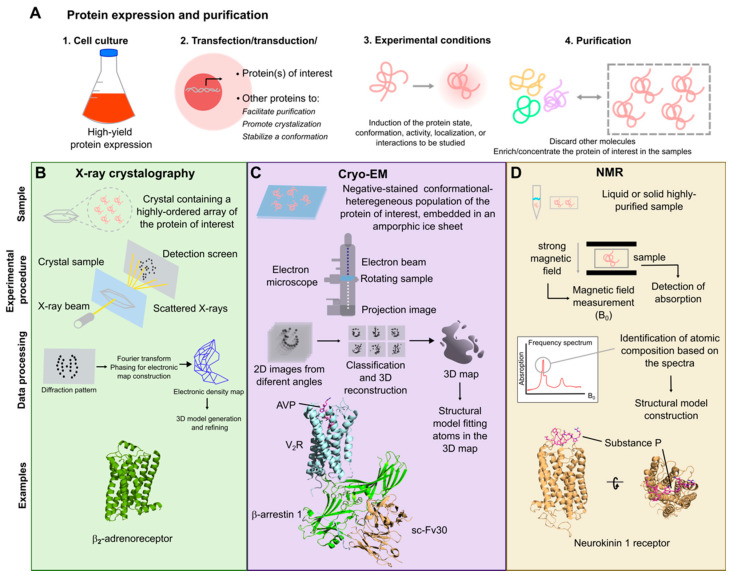
Experimental methods used to study protein structures. (**A**) Experimental structure biology requires large quantities of high-quality purified protein. Cell cultures (bacterial, insect, or mammal) are the most common systems used to express high amounts of protein, which is followed by various steps to isolate the protein of interest. (**B**) Schematic representation of how X-ray crystallography is applied to obtain high-resolution models of protein structures. The crystal structure of the β2-adrenoreceptor is presented here (adapted from PDB 2RH1). (**C**) Illustration of how high-resolution protein structures are determined using cryo-electron microscopy (cryo-EM). The V_2_R-βarr1 active complex structure presented here (PDB: 7DF9) is an example of a cryo-EM structure. (**D**) Representation of the nuclear magnetic resonance (NMR) spectroscopy workflow applied to study structure biology of peptides and proteins. The diagram shows the NMR-resolved structure of Substance P docked into a homology-modeled neurokinin 1 receptor (PDB: 2KS9).

Before any structural determination can begin, the protein of interest must be expressed in large quantities, isolated, and purified (Figure 2A). Although this appears straightforward, membrane proteins such as GPCRs are difficult to express and need to be solubilized in detergents where functionality can be lost [20]. In the case of X-ray crystallography, which was used to solve the first structures of GPCRs [21,22,23], purified protein is then mixed with chemical solutions under different conditions to crystallize the protein (Figure 2B) [20,24]. The formation of crystals can be difficult for proteins, making this process highly challenging and labor-intensive, especially for proteins that are glycosylated, contain flexible/less constrained domains, or are embedded in the membrane [24]. Another major challenge with GPCRs is that they adopt multiple conformations, and thus, need to be stabilized in a particular conformation to obtain high-resolution structural information. To this end, antibody-binder fragments can be developed that bind and stabilize GPCRs or transducers in a specific conformation [25,26,27,28,29]. In fact, early on, antibody-binder fragments/nanobodies were used to stabilize GPCRs in their inactive conformations when bound to antagonists/inverse agonists or active conformations when bound to agonists [23,29,30]. These efforts not only provided detailed insights into how ligands engage with GPCRs, but also into the structural changes that takes place in the receptor during its activation. Once crystals are obtained, X-ray beams are shot at the crystal and are scattered, producing a diffraction pattern that is captured on a detector plate. Based on this pattern, the protein electron densities can be examined and can help determine the 3D shape of the protein [27,31]. This technique usually works best with single proteins as opposed to protein complexes, and thus, several GPCR structures have been solved by X-ray crystallography and only a few complexes have been solved, such as the β_2_-adrenergic receptor–Gs complex and the rhodopsin–arrestin-1 complex [28,32].

One major disadvantage of using X-ray crystallography to solve GPCR structures is that GPCRs are difficult to crystallize due to low natural abundance, structural instability, and minimal polar surface area. To overcome these challenges, the receptor construct is often heavily mutated or fused with proteins that, together, enhance its expression and facilitate the crystallogenesis process. However, introducing such modifications might also result in a protein that differs significantly from the natural GPCR, and thus, structural artifacts might result. For these and other reasons, the field pivoted towards cryo-EM as the preferred method to solve GPCR structures over the past 5–8 years.

For cryo-EM, purified proteins are placed within a thin layer of flash-frozen amorphic ice on a metal grid and visualized at different angles under cryogenic temperature using powerful and very advanced electron microscopes (Figure 2C) [20,33]. Once thousands to millions of protein particles have been imaged, they are separated into distinct classes based on their appearance to generate high-resolution 2D images of the particle from different angles. Finally, multiple 2D classes are combined computationally to generate 3D models of the protein of interest [34,35]. A major advantage of cryo-EM over crystallography is that the complexes are imaged in an aqueous environment, and thus, their structures are obtained from an environment that resembles a more physiologically relevant one. Unlike the other methods described here, cryo-EM works best for larger proteins or protein complexes and does not have the same high requirement for large amounts of purified proteins [20,35]. For that reason, this technique has been most successful at solving structures of GPCR complexes including GPCR–G protein, GPCR–arrestin, GPCR–G protein–arrestin, GPCR–GRK, and GPCR–GRK–G protein complexes [36,37,38,39,40,41,42,43,44,45,46,47,48,49,50]. Currently, over 600 GPCR complexes have been solved almost exclusively by cryo-EM “https://gpcrdb.org/structure/statistics (accessed on 1 October 2024)”.

Finally, with NMR spectroscopy, a purified protein solution is placed in a strong magnetic field where different sequences of radiofrequencies are applied, and the resulting spectra can help provide information about the interactions between nuclei and their environment to calculate the protein structure (Figure 2D) [51]. However, this method typically works for smaller proteins, allowing them to be visualized effectively; as a result, this method requires a large amount of purified protein [20,51]. For these reasons, NMR has been applied to obtain high-resolution structures of peptide ligands and to study the conformational dynamics of GPCRs rather than to obtain high-resolution GPCR structures [52,53,54].

Regardless of the method, these experimental methods require extensive optimization and troubleshooting during most stages of the procedure before a final high-resolution protein structure can be obtained. These steps include protein expression, solubilization, purification, complex formation, protein crystallization, grid preparation, and data analysis, among others. Thus, experimental methods used for structural determination are expensive in terms of time and resources, and it is not guaranteed that a near atomic-level resolution structure of a particular protein will be achieved at the end.

## 4. Computational Approaches to Predict Structural Models

While the above-mentioned experimental methods remain the gold standard for protein structure determination and provide the most accurate results, computational approaches—most notably homology modeling and ab initio modeling—have emerged over recent decades [55]. These computational approaches have become essential tools for predicting protein structures in cases where experimentally derived structural data are impractical for use or are unavailable. The use of computational methods to predict protein structures can be especially valuable for drug discovery.

**Figure 3:**
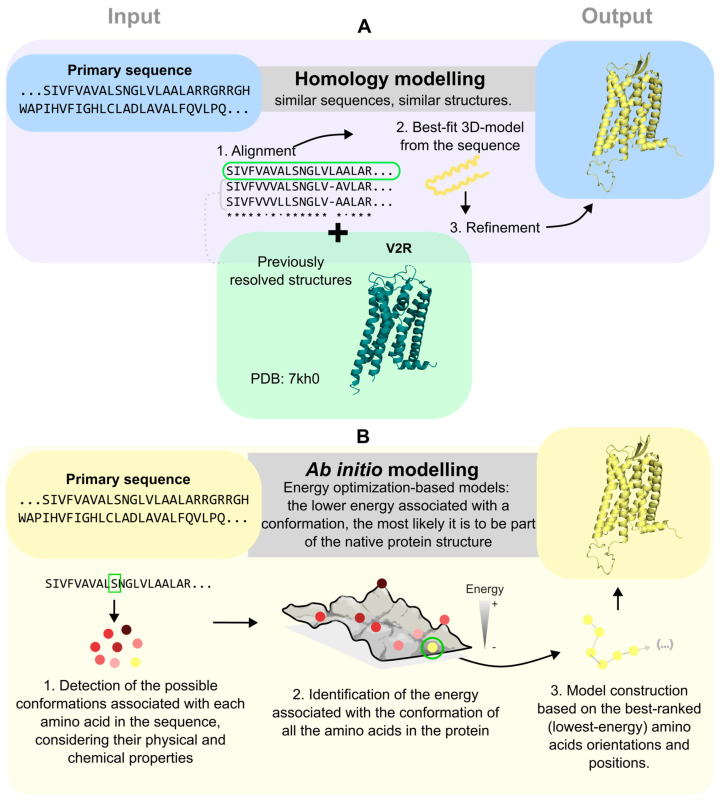
Computational methods used to obtain structural models. (**A**) Homology modeling relies on the biochemical principle that similar sequences lead to in similar structures. Following this principle, the protein sequence is aligned and compared to multiple sequences of other proteins from the same and different organisms. Then, the program searches for available structures in databases and constructs a new 3D model based on segments of other models with the similar arrays of amino acids. The final model is obtained through rounds of refinements that integrate physical and chemical considerations to finetune the location of each atom. A homology model made by Swiss-Model is presented, where the sequence of V_2_R is used as the input and PDB 7KH0 as the template. (**B**) Ab initio modeling generates 3D structures from “scratch” without the need for sequences associated with previously resolved structures. It considers the physical and chemical properties of each amino acid in the sequence and calculates possible structural conformations of the protein with their associated energies. Finally, a structural model is generated with the lowest possible conformational energy based on the principle that conformations with lower energy are more stable, and thus, more likely to exist.

The SWISS-MODEL is one of the first computational, automated web-based tools dedicated to protein structure homology modeling; this modeling server allows users to generate 3D models of proteins based on their amino acid sequences and is the automated modeling system that has been most widely used over the past decade [56]. The model is based on the idea that proteins with similar sequences have similar structures [56]. Therefore, using the SWISS-MODEL, users input a target protein sequence and the system automatically identifies a suitable template structure that aligns with the given target sequence from a database (i.e., Protein Data Bank) selecting those with sequences that are similar to the target protein (Figure 3A) [56,57]. Based on this alignment, a 3D model of the target protein can be constructed (Figure 3A) [56,57]. From the database templates, the system generates a model of the target protein that can be used for further studies. The model undergoes further refinement, particularly in regions where the target protein significantly differs from the template, such as in loop regions (Figure 3A) [56]. These loop regions are often the most variable and flexible parts of proteins and are particularly challenging to model accurately without any experimental data to base the structure off. However, despite this challenge, loop refinement is critical for the accurate prediction of protein–ligand interactions, which are often influenced by these regions [56]. Nonetheless, the final output is a 3D model of the target protein, which has been valuable in drug discovery, protein function, and protein binding predictions [56,58].

Another homology modeling approach that has been applied to GPCRs is MODELLER. MODELLER is similar to SWISS-MODEL in its approach, leveraging known protein structures to model target proteins. However, it offers additional flexibility in terms of handling more complex alignments. Thus, due to its flexibility and ability to target multi-template modeling, MODELLER has been a popular method for predicting structural models of diverse receptors, including 5-HT_2A_ receptor, 5-HT_5A_ receptor, GPR68, and MRGPRX2, which successfully have been used for ligand discovery [59,60,61,62]. For these example GPCRs, the sequences of the target GPCR, the closest homolog with a known structure, and additional homologs were aligned using multiple sequence alignment. The alignments were manually edited to remove any additional amino acids in the template sequence that exceeds the target protein sequence, or domains that were engineered into the template sequence for structural elucidation purposes, such as apocytochrome b562 RIL (BRIL) and T4 lysozyme. Between 500 and 1000 homology models were calculated for each of the target GPCRs from the structures of the closest homologs, preferably bound to an agonist to mimic the active conformation at the orthosteric site of the model structure, through iterative rounds of model refinement using MODELLER. The models were then subjected to validation though virtual docking against known agonists and antagonists of the target GPCRs and ranked by their ability to enrich the ligands. The best ranked models were further optimized by energy minimization.

Although homology modeling is an effective way of predicting models for structure-based drug discovery, its success is contingent on the availability of suitable structure templates. Thus, the reliability of models may be questionable when no closely related protein structures are available. In such cases, ab initio modeling is an alternative computational method that has been used to predict 3D structures of proteins from their amino acid sequences. Unlike homology modeling, ab initio modeling does not rely on databanks and known protein structures to predict structures, but rather by using basic physical principles of protein folding and amino acid interactions (Figure 3B) [63]. In this way, ab initio modeling can automatically compute different structures by exploring a vast amount of different information that the amino acid chain can adopt (Figure 3B) [63]. The method predicts the most likely structural model by choosing the one that has the lowest free energy in its native environment (Figure 3B) [64]. Ab initio modeling has emerged as a type of computational power through its use of simple chemical and physical modeling techniques and algorithms. Although ab initio modeling performs well for smaller simple proteins, its usefulness can be limited specifically for larger proteins, including GPCRs, due to their longer and more complex amino acid chains and all the different possible conformations that can be made from a single strand of amino acids [65].

## 5. Structure Biology and AI/ML

An emerging computational approach for predicting structural models of proteins is via AI/ML. One of the best-known examples of AI usage for structure prediction is AlphaFold, a program developed by the British–American Artificial Intelligence research laboratory, DeepMind (London, England). AlphaFold first gained attention in 2018, when it won the 13th Critical Assessment of Protein Structure Prediction (CASP13), a biennial competition for protein prediction accuracy among different methodologies [66].

Two years later, AlphaFold2 won the subsequent CASP14 competition with a much larger margin than the previous version. In fact, AlphaFold2 was much more accurate than its closest competitors and was able to produce highly accurate domain structures and side chains than traditional template-based methods [67]. To date, AlphaFold2 has been used to predict the structures of more than 200 million proteins from over 1 million species [67,68].

**Figure 4:**
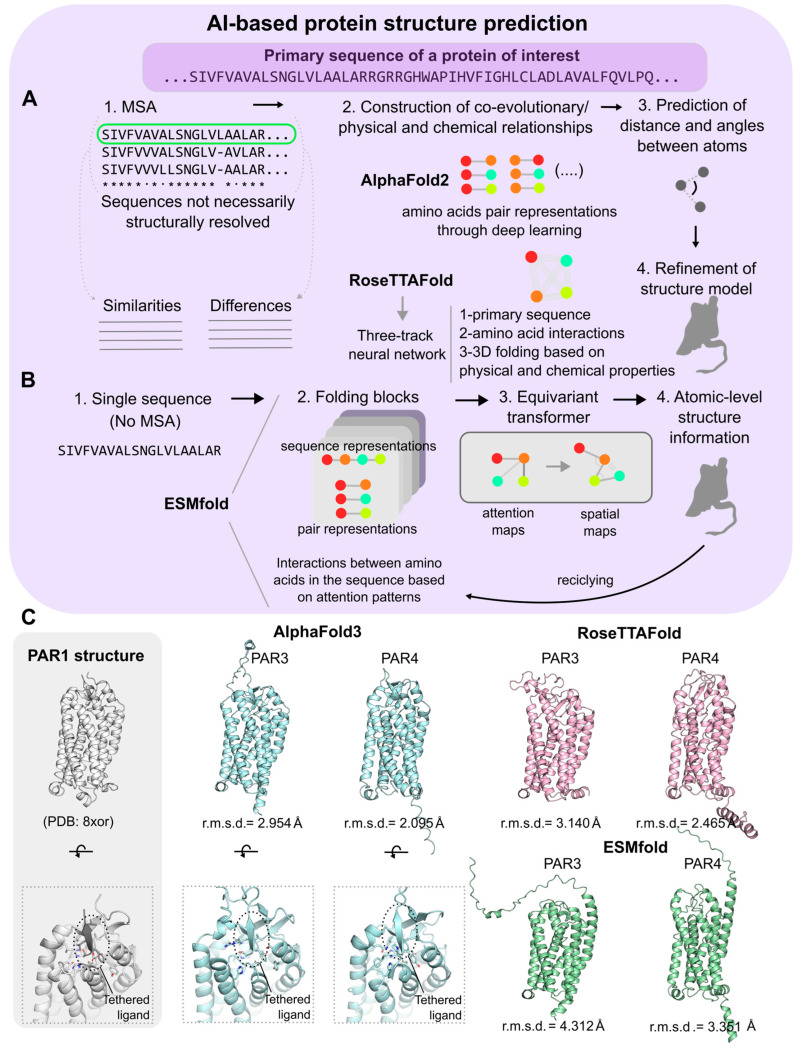
AI-based structure prediction applications. (**A**) Based on multiple sequence alignments (MSA), artificial intelligence (AI) platforms detect differences and similarities between amino acids from several related sequences. This information is used to construct evolutionary relationships between the amino acids at specific positions, which is applied to determine their relative positions within the protein of interest. AlphaFold2 and RosettaFold are two of the best-performing AI platforms that predict new structural models using these principles. (**B**) ESMfold is an AI platform that does not require MSA, which speeds up the processing time and reduces the computing costs of protein structure predictions. It was trained using a large language model that masked random amino acids from millions of evolutionarily distinct sequences to recognize patterns within the sequences. Using a series of folding blocks, it generates attention maps from an amino acid sequence of interest that is then transformed into a spatial map with atomic resolution. (**C**) Structural models of protease-activated receptors 3 (PAR3) and 4 (PAR4) generated with AlphaFold, RoseTTAFold, and ESMfold. As no structures of these receptors exist, their similarities to the experimentally solved and closely related PAR1 structure (PDB ID: 8xor) were assessed by calculating the r.m.s.d. values between them. In addition, PARs are unique in that their natural peptide ligands are part of the N-terminals themselves but are hidden away by other parts of these domains in their inactive forms. These tethered ligands are only exposed when proteases cleave and remove the inactivating part of the N-terminals of PARs. In this example, the PAR3 and PAR4 structures were predicted with the tethered ligands exposed. Interestingly, only AlphaFold3 was able to predict the binding of these tethered ligands to their orthosteric binding sites within the respective receptor.

At the core of AlphaFold2’s success is its sophisticated deep learning system based on algorithms that recognize specific patterns, which allow for complex computations; the system controls and analyzes both physical and evolutionary data, which are trained on datasets of known protein structures (primarily from the Protein Data Bank), while enhancing and refining the accuracy of the predicted proteins using deep learning techniques and bioinformatics to create extremely accurate and reliable predictions [67]. The deep learning system uses an advanced neural network known as a transformer to help interpret the relationship between amino acid residues in a protein sequence. The transformer architecture, equipped with attention mechanisms, allows AlphaFold2 to analyze the entire amino acid sequence of a protein in parallel via multiple sequence alignment (MSA) analysis to identify associations between amino acids that are most important for the protein’s structure/function (Figure 4A) [67,68]. However, the system goes beyond using raw data and the structure module integrates both physical and biological aspects of protein structures such as bond angle/geometry, structural protein motifs, and amino acid interactions to help guide the model toward an accurate protein prediction (Figure 4A) [67,69].

With the established learning architecture and training model, AlphaFold2 can be used to predict the distances between amino acids and the angles between their bonds to process and predict the correct protein folding using these spatial relationships [70]. Once this information has been processed and the initial predictions have been made, AlphaFold2 refines the initial structure iteratively, adjusting the distances and angles to create a better alignment that agrees with known physical and biological constraints that govern how normal proteins fold [64,70]. The program continues to adjust the distances and angles of bonds to minimize binding constraints to ultimately achieve a structure that aligns with an accurate and feasible protein prediction with higher efficiency and fewer resources. In addition to the 200 million predictions of single protein structures [71], which include all GPCRs and from multiple species, AlphaFold2 has also been applied to predict state-specific conformations of all human non-olfactory GPCRs [72].

Another computational method that utilizes deep learning for predicting protein structures is RoseTTAFold, which was developed by the Baker lab at the University of Washington and was the runner-up to AlphaFold2 in CASP14 [72]. RoseTTAFold also predicts protein structure with high accuracy using MSA bioinformatics but is distinct from AlphaFold2 in its approach. Unique to RoseTTAFold is its “three-track” neural network architecture that integrates the prediction of both secondary and tertiary structures of proteins (Figure 4A). This specialized deep learning system processes amino acid sequences through distinct yet interconnected pathways, each of which are dedicated to analyzing a different aspect of the protein data [73]. The 1D track focuses on the amino acid chain, the 2D track interprets the spatial relationships between the amino acids necessary for protein folding, and the 3D track provides more detailed information about the atomic position that contributes to the protein’s overall structure. These three outputs are then analyzed and processed together to generate a comprehensive and highly accurate 3D model of the protein. By processing these layers simultaneously, RoseTTAFold offers a more nuanced and detailed analysis of the protein structures, which in turn enables a more accurate prediction of how proteins fold [73,74].

In a comparative study focusing on GPCRs, both AlphaFold2 and RosettaFold were evaluated, and the results showed that AlphaFold2’s average minimum distance (five-model minimum) and root mean square deviation (r.m.s.d.) of the top-scored model were better than RosettaFold’s; this revealed that the overall prediction algorithms were more accurate for AlphaFold2 compared to RoseTTAFold [72]. However, RoseTTAFold had a smaller average variance in its model predictions, suggesting that while AlphaFold2 might produce more accurate top models, RosettaFold provides more consistently reliable predictions across multiple different models [72,74]. Regardless, both AlphaFol2d and RoseTTAFold have revolutionized the field of protein structure prediction, offering a more comprehensive understanding of the complex world of proteins. Very recently, a new version of RoseTTAFold, RoseTTAFold2, has been made available [73,74]. RoseTTAFold2 uses features both from the original RoseTTAFold, such as the three-track neural network system [73], but also from AlphaFold2, including the use of a distillation set. The resulting algorithm performs as AlphaFold2 on monomers and complexes, with better computational scaling for large proteins and complexes [74].

Another AI-based approach to structure prediction involves large language models. The models have been trained on available sequences in databases to learn evolutionary, structural, and functional patterns to predict structures from input amino acid sequences. One of these programs is ESMFold, which utilizes a trained transformer protein language model with 15 billion parameters that captures and leverages evolutionary patterns in protein sequences to produce accurate structural predictions (Figure 4B) [75]. Unlike AlphaFold2 and RoseTTAFold, ESMFold does not rely on MSA and uses a greatly simplified neural architecture for its interface. These differences greatly improve the speed of the interference forward pass by up to 60× while also removing the search process for related protein sequences, which is a time and computational resource-heavy process [75]. The net result is a significantly faster structure prediction than AlphaFold2 and RoseTTAFold, but in terms of accuracy, ESMFold lags slightly behind.

Although these new AI/ML applications are impressive in terms of their ability to accurately predict structural protein models, caution should be used when interpreting the results. This is particularly true when assessing the structural effects of mutations and when predicting hypothetical protein complexes. It is important to emphasize that the predictions are only models, and that one should always seek to validate them experimentally.

## 6. Computation-Based Docking/Virtual Screening

**Figure 5:**
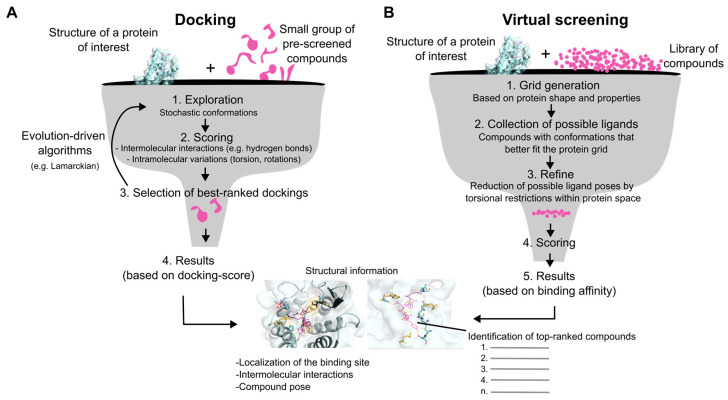
Docking and virtual screening: computational tools for studying ligand–target interactions. (**A**) During molecular docking, ligands are computationally fitted into potential binding pockets of the protein structural model, which will suggest (1) compounds that potentially interact with the protein, and (2) specific chemical bonds that make up the ligand–target interaction. (**B**) Virtual screening follows the same basic principles as molecular docking. However, it aims to screen a library of thousands or even millions of compounds against a particular binding pocket of the target protein, which is defined by a pre-selected grid space. The output of a virtual screening is a list of possible candidates of ligands, which can be further analyzed by traditional docking or experimental assays.

Beyond providing valuable insights related to protein function, structural models also offer a map of potential binding pockets that can be targeted by small-molecule compounds, peptides, and other molecules. The coordinate grids of these potential binding sites can be used to computationally “dock” ligands into them (Figure 5A). Docking programs use algorithms to predict and test multiple orientations and binding positions of the ligand to the binding pocket while calculating other factors such as hydrogen bonding, hydrophobic interaction, and electrostatic interactions of the ligand–target complex [76,77,78]. Ultimately, each conformation is then scored based on how well the molecule fits, which is commonly known as a docking score. Some of the most popular docking programs include AutoDock, Glide, and GOLD49 [78,79,80], but many additional programs exist.

Virtual screening refers to a docking-based technique in which large libraries of molecules are docked into a binding pocket of the protein of interest to determine which of them are most likely to be ligands (Figure 5B) [81]. This is particularly helpful for determining which ligands are good candidates for further drug testing and helps research within drug discovery. While regular docking aims to understand the specifics of ligand–target binding and the different binding of a few ligands, virtual screening aims to filter a larger library dataset of ligands to a smaller subset of promising ligand candidates for target protein engagement for further study and research [82].

As with regular docking, a long list of virtual screening programs exist that differ slightly. In respect to GPCRs, one of the most applied programs is called DOCK and has successfully been used to virtually screen and discover ligands for several receptors, including μ-opioid receptor, the atypical opioid receptor MRGPRX2, D4 dopamine receptor, acetylcholine M2 receptor, melatonin receptors, 5-HT_2A_ receptor, and the β_2_-adrenergic receptor [60,61,62,83,84,85,86,87,88]. Since a comprehensive review on the examples of molecular docking/virtual screening on GPCRs was conducted by Ballante et al. in 2021 [76], there have been additional reports of successful screens against receptors such as β_3_-adrenergic receptor, CC chemokine Receptor 7, free fatty acid receptor 4, metabotropic glutamate receptor 5, protease-activated receptor 4, trace amine-associated receptor 1, GPR34, GPR52, GPR55, GPR78, GPR88, and GPR139 [89,90,91,92,93,94,95,96,97,98,99,100]. In contrast with the previous review, structures of more than half of these receptors could be predicted either by MODELLER, SWISS-MODEL, or AlphaFold2 with the recent advances. Additionally, the structures were screened not only against the ZINC database [101], but other smaller, more specialized libraries including ChEMBL, ChemDiv, DrugBank, Enamine, FKKTLib, or Specs database [102,103,104]. Screens against CCR7 and mGlu_5_ were performed to exclusively target allosteric sites instead of their orthosteric sites to improve the receptor subtype selectivity of the ligands. All the screens have successfully discovered agonists or antagonists of the receptors, which had half maximal effective concentrations (EC_50_) or half maximal inhibitory concentrations (IC_50_) in the sub-micromolar range.

However, while they provide valuable insights into the molecular basis of drug–target interactions, which is critical for understanding drug action and designing better drugs, the accuracy of these docking methods depends heavily on the quality of the protein structure and the algorithms used for docking and scoring [105]. Ultimately, since they are based on algorithmic structures and computational methods to determine binding, they cannot fully capture biological systems’ dynamic and complex nature. Thus, the computational-based docking/virtual screening should be considered as hypothesis generators, where a substantial portion of the predicted ligand–target interactions are false while a few of them are correct, rather than actual experimental data.

## 7. AI-Enabled Structure-Based GPCR Drug Discovery: Are We There Yet?

Despite the challenges posed by the dynamic nature of GPCRs, advancements in computational techniques continue to enhance our understanding of these critical proteins. For example, the use of AI in structural biology and protein prediction has opened up new avenues in drug discovery. Recent studies focusing on the accuracy of AlphaFold2 models for docking-based drug discovery have compared these AI-generated protein models with traditional experimental PDB structures in the use of computational docking algorithms [106]. These studies have consistently found that while AlphaFold2’s models excel in predicting protein architecture, they often fall short in docking applications [106]. This discrepancy underscores the need for possible further refinement of AlphaFold2 protein models and further advancements and improvements post-prediction to ensure their reliability and future use in high-throughput docking applications. For example, a recent trial compared AlphaFold2-generated structural models of GPCRs with traditional template-based models and experimentally determined structures [106]. Interestingly, the authors found that while AlphaFold2’s models excelled in predicting ligand binding pocket structures as compared to traditional template-based models, they fell short in their usage in predicting the ligand pose by docking as compared to experimentally solved structures [106]. It was noted that although the overall structures of the AlphaFold2 models were accurate, the exact position of the side chains of the amino acid residues that make up the ligand binding pocket were somewhat out of position, which could affect ligand binding predictions.

Others have noted similar inaccurate positions of side chains in AlphaFold2 predictions, and some predictions that are deemed to be of high confidence do not correspond well with structures that have been obtained experimentally [107]. Most recently, it was found that ligands of the β_2_-adrenergic receptor and 5-HT_2A_ receptor identified by docking against crystal and cryo-EM structures, respectively, docked poorly against corresponding AlphaFold2 models [108]. However, the authors suggested that a potential flaw in the retrospective logic of docking known ligands against known structures is that the ligands chosen are biased by the receptor structure, and the receptor conformation is adapted to the ligands with which it was determined. As GPCRs are highly dynamic proteins that can adopt several active conformations, the failure of docking known ligands into a hypothetical receptor conformation predicted by AlphaFold2 does not exclude that (1) the predicted receptor conformation exists, and (2) that new unknown ligands fit into the AlphaFold2-predicted binding pocket. Therefore, the investigators compared the performance of virtual screening against the β_2_-adrenergic receptor and 5-HT_2A_ receptor respective crystal and cryo-EM structures with the structural models predicted by AlphaFold2. Surprisingly, using this prospective approach, the crystal/cryo-EM structures performed almost identically to the AlphaFold2 models in discovering new ligands against the β_2_-adrenergic receptor and 5-HT_2A_ receptor [108]. These results highlighted the potential usefulness of AlphaFold2 and other prediction programs in early drug discovery.

## 8. Conclusions

While writing this review, new AI-based applications have been released that not only predict the structure of a given protein of interest with high accuracy but also can predict how small-molecule ligands engage with it. These programs include AlphaFold3, RoseTTAFold All-Atom, and Neuralplexer [109,110,111]. In addition, AlphaFold3 and RoseTTAFold All-Atom expanded the capabilities of their previous algorithms by also allowing structural predictions of protein complexes with other biomolecules such as DNA, RNA, and ions [109,110]. The overall network architectures of these programs resemble those of their previous versions with some key differences. For example, RoseTTAFold All-Atom uses the original three-track neural network approach to predict structural complexes of biomolecules, whereas small molecules are introduced in the system as atomic-bond graphs [109]. AlphaFold3 still relies on MSA processing, although to a significantly lesser degree, and has replaced the original structure module for a new diffusion module [110]. The new and approved system predicts protein–protein complexes with higher accuracy than previous AlphaFold versions and predicts protein–nucleic acids complexes with higher accuracy than other leading programs [110]. Despite these improvements, the release of these latest applications has been subject to controversy, as some of their source codes were not released together with their publications, and in some cases, they were not even available for the referees to review (the source codes for AlphaFold3 have since been released) [112,113,114]. In addition, the part of these applications involving small molecule–target interactions were not initially available to the public for testing, something that has been noticed and criticized in the field. These traits are signs of a rapidly evolving and fiercely competitive field, which will undoubtedly revolutionize how drug discovery is conducted. The importance and popularity of the field was most recently acknowledged by awarding the Nobel Prize in Chemistry to key individuals responsible for the development of AlphaFold and RoseTTAFold. In terms of GPCRs, few publications have documented the usefulness of AI-enabled structure-based drug discovery, but stories of how these techniques are being applied to discover new GPCR ligands are simmering under the surface. Thus, we “predict” that these approaches will dominate GPCR drug discovery in the future.

## Data Availability

Not applicable.
